# Recent Advances in Quantum Dots for Photocatalytic CO_2_ Reduction: A Mini-Review

**DOI:** 10.3389/fchem.2021.734108

**Published:** 2021-09-30

**Authors:** Young Ho Park, G. Murali, Jeevan Kumar Reddy Modigunta, Insik In, Su-Il In

**Affiliations:** ^1^ Department of Polymer Science and Engineering, Korea National University of Transportation, Chungju, South Korea; ^2^ Department of IT-Energy Convergence (BK21 FOUR), Chemical Industry Institute, Korea National University of Transportation, Chungju, South Korea; ^3^ Department of Energy Science and Engineering, Innovative Materials and Devices for Future Electronics/Power Sources (BK21 FOUR), Daegu Gyeongbuk Institute of Science and Technology (DGIST), Daegu, South Korea

**Keywords:** CO_2_ reduction, perovskite, MXene, quantum dots, carbon quantum dot, transition metal chalcogenide, metal oxide, photocatalyst

## Abstract

Solar energy–driven carbon dioxide (CO_2_) reduction to valuable solar fuels/chemicals (e.g., methane, ethanol, and carbon monoxide) using particulate photocatalysts is regarded as one of the promising and effective approaches to deal with energy scarcity and global warming. The growth of nanotechnology plays an eminent role in improving CO_2_ reduction (CO_2_R) efficiencies by means of offering opportunities to tailor the morphology of photocatalysts at a nanoscale regime to achieve enhanced surface reactivity, solar light absorption, and charge separation, which are decisive factors for high CO_2_R efficiency. Notably, quantum dots (QDs), tiny pieces of semiconductors with sizes below 20 nm, offering a myriad of advantages including maximum surface atoms, very short charge migration lengths, size-dependent energy band positions, multiple exciton generation effect, and unique optical properties, have recently become a rising star in the CO_2_R application. In this review, we briefly summarized the progress so far achieved in QD-assisted CO_2_ photoreduction, highlighting the advantages of QDs prepared with diverse chemical compositions such as metal oxides, metal chalcogenides, carbon, metal halide perovskites, and MXenes.

## Introduction

**FIGURE 1:**
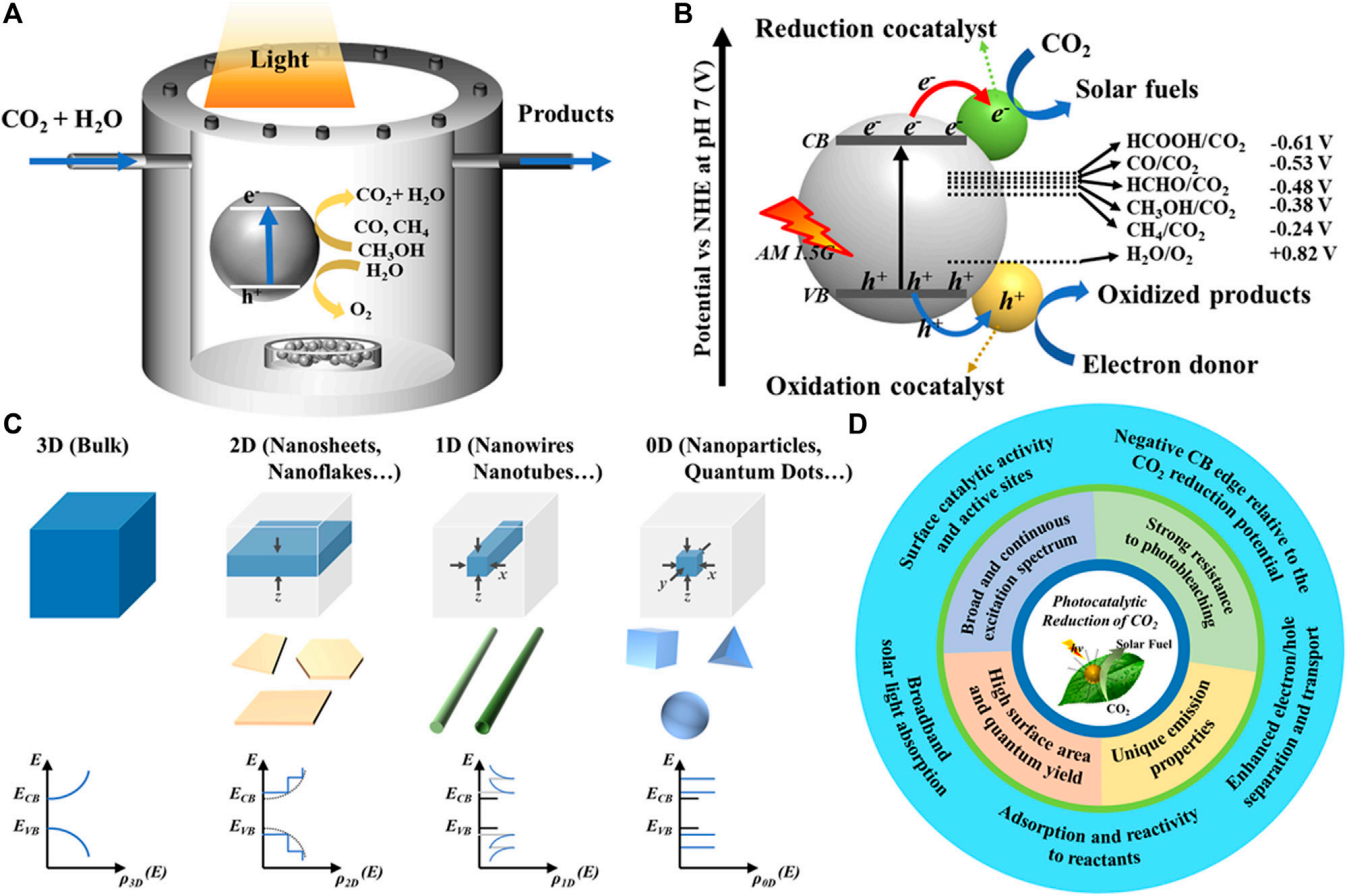
Schematic illustration of the **(A)** photocatalytic CO_2_ conversion method, **(B)** CO_2_ conversion by semiconducting photocatalyst, **(C)** density of states modification under different degrees of quantum confinement, and **(D)** the advantages of the QDs for the photocatalytic reduction of CO_2_.

Carbon dioxide (CO_2_) is the major constituent of the global warming gases that are destroying the ozone layer of the Earth. Many researchers have been trying to capture and convert greenhouse gases, especially CO_2_, to make it as a pollution-free and recyclable energy source. In the present era, CO_2_ has been captured (Ibrahim et al., 2018; Fu et al., 2019; Omodolor et al., 2020; Dhoke et al., 2021; Lau et al., 2021), converted (Fu et al., 2019; Omodolor et al., 2020) and stored (Lau et al., 2021) by using different technologies. There are many methods and techniques that are studied for the conversion of CO_2_ into renewable energy sources; among them, photocatalytic CO_2_ reduction (CO_2_R) (Sorcar et al., 2018; Sorcar et al., 2019; Albero et al., 2020; Li et al., 2021), electrochemical CO_2_R (Jia et al., 2019; Liang et al., 2020), photo-biochemical CO_2_R (Kim et al., 2018), photo-electrochemical CO_2_R (Roy et al., 2016), and thermochemical CO_2_R (Maiti et al., 2018; Pullar et al., 2019) are well-known (He and Janáky, 2020). The photocatalysis process promotes the conversion reactions using clean solar energy, which is an eco-friendly CO_2_ conversion technology (shown in Figure 1A).

The photocatalytic CO_2_R reaction comprises three primary steps: 1) the semiconductor photocatalyst absorbs the solar light energy and generates photocharge carriers, 2) photogenerated charge carriers were separated and transported to the surface of the semiconductor photocatalyst, and 3) oxidation and reduction reactions mediated by holes and electrons take place at the active sites on the photocatalyst surface, that is, electrons for CO_2_R and holes for oxidation of sacrificial agent or water, respectively (Xie et al., 2016; Wu et al., 2019) (Figure 1B). However, to exhibit the CO_2_ photoreduction, the photocatalyst should have the ability to adsorb CO_2_ and must possess its valence band (VB) at more positive potential than the water oxidation potential and conduction band (CB) at more negative potential than the CO_2_R potential (Xie et al., 2016). It should be noted that CO_2_R into CO_2_^.─^ radicals through single electrons transfer is unfavorable to occur because of the required high negative potential for the electrons in the CB of photocatalyst (−1.9 V *vs* NHE) (Shit et al., 2020). However, owing to relatively lower negative potential required for the conversion of CO_2_ into hydrocarbons, the proton-assisted multielectron transfer process is more favorable (Shit et al., 2020). Depending on the number of participated electrons, various gas and liquid phase hydrocarbons, such as carbon monoxide (CO), formic acid (CH_2_O_2_), oxalic acid (C_2_H_2_O_4_), formaldehyde (CH_2_O), acetaldehyde (C_2_H_4_O), methanol (CH_3_OH), methane (CH_4_), ethylene (C_2_H_4_), and ethanol (C_2_H_5_OH), are produced in the CO_2_R reaction (Shit et al., 2020) (Figure 1B). Major concerns existing for the efficient photocatalytic CO_2_R are 1) photocatalyst materials’ limited light absorption ability, 2) quick recombination of photogenerated charge carriers, and 3) poor adsorption of CO_2_ molecules on the photocatalyst surface. It was realized that crystalline phases, size, shape, and exposed facets of photocatalyst are crucially influencing the CO_2_R. Maneuvering semiconductors into distinct nanostructures results in significantly altered surfaces and electronic structures, which affect the surface reactivity and positions of energy bands, respectively (Figure 1C). Furthermore, the morphological features are also pivotal for the transfer of photocharge carriers. For instance, 2D nanosheet morphology having one dimension in the atomic thickness level facilitates shorter charge migration lengths, which is beneficial to avoid the quick recombination kinetics. Furthermore, their flat surface allows the facile heterojunction formation with other 2D, 1D, and 0D nanostructures. However, the quantum confinement in three dimension leaves 0D quantum dots (QDs) with very short charge migration lengths and maximum surface-exposed atoms (Figure 1C). The higher sensitivity of energy band positions to the size of QDs allows the precise tailoring of their VB and CB to the required positions in order to initiate the reduction of CO_2_ into selective hydrocarbons. Furthermore, the tiny size allows their easy grafting on other 2D and 1D nanostructures to frame the heterojunctions. Hence, QDs of a variety of semiconductors having several advantages garnered significant attention for the photocatalytic CO_2_R applications (Figure 1D).

### Metal Oxide Quantum Dots

Metal oxide semiconductor materials such as MgO (Kohno et al., 2001), ZrO_2_ (Hengne et al., 2018; Miao et al., 2019), ZnO (Gokon et al., 2003), WO_3_ (Jin et al., 2015), and TiO_2_ (Kočí et al., 2009; Yu et al., 2014) have been studied as catalysts and co-catalysts for the photocatalytic reduction of CO_2_. In order to enhance the photocatalytic reduction of CO_2_, 0D metal oxide QDs (MOQDs) have been studied apart from their bulk counterparts due to their advantages like economical, eco-friendly, high surface area, good dispersibility, and well-maintained light absorption ability. For instance, the activity of CuO QDs is in good compatibility with Ti in the metal organic framework (MOF) MIL-125 coupled with g-C_3_N_4_ toward the efficient photocatalytic CO_2_R to form CO, CH_3_OH, CH_3_CHO, and C_2_H_5_OH (Li et al., 2020). The good compatibility between CuO QDs and active sites of Ti in MIL-125 the electrons generated by photocatalytic activity will easily transfer to CuO from MIL-125/g-C_3_N_4_. The combination of g-C_3_N_4_/CuO on MIL-125 has drastically improved the yield of CO, CH_3_OH, CH_3_CHO, and C_2_H_5_OH in the presence of water. However, most of the MOQDs have some unresolved technical issues such as the low yield of available electrons and large intrinsic bandgaps that are restricting the wide range applicability under visible light irradiation (Heng et al., 2021). Introduction of defects, doping, and heterojunction formation are the commonly practicing strategies to improve the CO_2_R efficiency of MOQDs.

### Transition Metal Chalcogenide Quantum Dots

Transition metal chalcogenide (TMC) materials are formed by the combination of IV-VII transition metal elements (Mo, W, V, Nb, Ta, Ti, Zr, Hf, Tc, or Re) and chalcogens (S, Se, or Te). By the controlled synthesis of the TMCs from bulk to 2D nanosheets or 0D QDs, the bandgaps in TMCs can be tuned with respect to size and shape (Yao et al., 2019; Pandey et al., 2020). There are more than 40 kinds of TMCs available till date, which can be synthesized in large quantities by using synthesis techniques such as the CVD method (Bosi, 2015; Severs Millard et al., 2020), hydrothermal method (Chen and Fan, 2001), and Langmuir–Schaefer deposition method (Kalosi et al., 2019).

The TMCQDs and their composites such as CdS (Kuehnel et al., 2017), CdS/Ni (Wang et al., 2010), CdSe/TiO_2_ (Sarkar et al., 2016), PbS (Wang et al., 2011), ZnS/CuInS_2_ (Lian et al., 2018), and Mn:CdS/CdSeTe/TiO_2_ (Nie et al., 2018) have proven to be effective performing photocatalysts. Wang et al. reported the heterostructured catalyst CdSe/Pt/TiO_2_ for the photoreduction of CO_2_ under visible light in the presence of water (Wang et al., 2010). CdSe QD-sensitized TiO_2_ heterostructure materials are capable of catalyzing CO_2_R under visible light illumination (*λ* > 420 nm). The CdSe QD’s surface was modified by removing surfactant caps through annealing and using a hydrazine reducing agent, which enhanced the direct contact between CdSe QDs and TiO_2_. Although TMCQDs show good performance, they slowly become inactive after continuous exposure to the visible light illumination, which is a commonly observed issue in TMC(QD)s due to gradual oxidation of TMCs. The surface stoichiometry of the TMCQDs influences the exciton kinetics such as in CdSe QDs, the presence of a higher surface ratio of Se increases the possibility of electron–hole recombination at trap sites. The surface stoichiometry manipulation drives effective ways to improve the photocatalytic performance of TMCQDs.

### Carbon Quantum Dots

Carbon QDs (CQDs), with their sizes in the range of 20 nm, have attracted much attention for their photoluminescence properties and co-catalyst role in different photocatalytic reactions. They exhibit low toxicity, good chemical stability, and exceptional water solubility compared to widely used semiconductor photocatalysts (CdS, TiO_2_, etc) (Murali et al., 2021). Importantly, CQDs possess upconversion photoluminescence property that allows the utilization of NIR light. All the aforementioned features and the high CO_2_ adsorption characteristics make CQDs an auspicious candidate for the photocatalytic CO_2_R application. Furthermore, surface functionalization with different organic functional groups tailors the semiconducting property and bandgap of CQDs to make them most suitable for CO_2_R. The functionalization of CQDs with 1,1′-bi(2-naphthylamine) enables the formation of intramolecular Z-scheme with a narrow bandgap for the efficient CO_2_R under visible light (Yan et al., 2018). Combining CQDs with other semiconductors is reported to enhance the CO_2_R efficiency by utilizing broad range of solar energy, where CQDs absorb visible light that enables the transfer of photogenerated charge carriers through the interface for efficient charge separation and improved CO_2_R. Specifically, heteroatom (N, B, S, Cl, etc.)-doped CQDs are more suitable to form the heterojunction owing to their enhanced light absorption, electron transport, chemical activity, and specific surface area properties. For instance, the N-rich CQDs/TiO_2_ composite showed an enhanced performance for the CO_2_R with CH_4_ and CO yield of 7.79 and 7.61 times higher than that of pristine TiO_2_ (Li et al., 2018).

### Perovskite Quantum Dots

**FIGURE 2:**
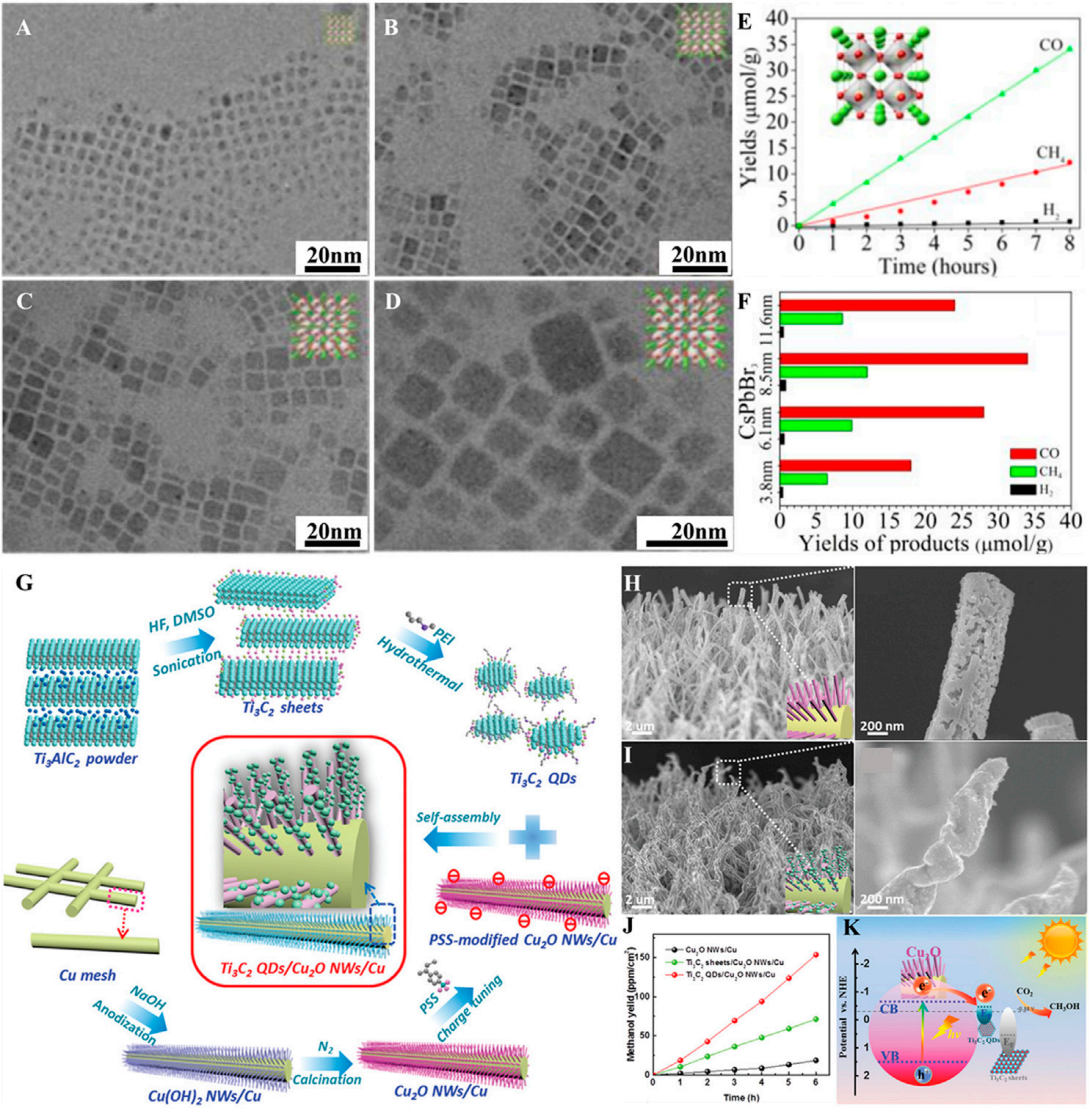
TEM images of CsPbBr_3_ QDs with particle sizes of **(A)** 3.8 nm, **(B)** 6.1 nm, **(C)** 8.5 nm, and **(D)** 11.6 nm (inset crystal structures). Photocatalytic CO_2_R for QDs with **(E)** 8.5 nm CsPbBr_3_ QDs and **(F)** CsPbBr_3_ QDs of different sizes (reproduced with permission from (Hou et al., 2017)). **(G)** Schematic illustration for synthesis of Ti_3_C_2_ QDs and Ti_3_C_2_ QDs/Cu_2_O NWs/Cu heterostructure, FE-SEM images of **(H)** Cu_2_O NWs/Cu, (i) Ti_3_C_2_ QDs/Cu_2_O NWs/Cu heterostructures, **(J)** CH_3_OH yield as a function of time, and **(K)** energy level diagram of Ti_3_C_2_ QDs/Cu_2_O NWs/Cu and Ti_3_C_2_ sheets/Cu_2_O NWs/Cu heterostructures (reproduced with permission from (Zeng et al., 2019)).

Metal halide perovskites are a class of semiconductors having ABX_3_ chemical stoichiometry, where A represents the alkali (e.g., Cs) or organic (e.g., formamidinium or methylammonium) cation; B denotes the divalent metal cation such as Pb, Bi, or Sn; and X stands for halide anions such as Cl, Br, or I. QDs of these materials are familiar for their excellent optical and electrical properties including strong light absorption, charge carrier’s high mobility and long diffusion lengths, and prolonged charge carrier lifetimes. The tuning of the cation/anion composition could facilitate the tailoring of the perovskite QD (PQD) absorption from UV to the NIR region (Shyamal and Pradhan, 2020). Furthermore, the favorable VB and CB positions of these CQDs enable the utilization of photogenerated charge carriers for CO_2_R prior to their recombination. However, selection of appropriate solvent for the photocatalytic CO_2_R over PQDs is a difficult task due to their instability upon exposure to polar solvents. Solvents like ethyl acetate have been selected because their mild polarity protects PQDs and CO_2_ is highly soluble in them (Xu et al., 2017). The addition of water to this solvent has been demonstrated to increase the selectivity of CO_2_R by minimizing H_2_ production (Shyamal and Pradhan, 2020). But an excessive amount of water addition will have negative impact on the stability of PQDs (Hou et al., 2017). However, the careful surface protection of cobalt-doped CsPbBr_3_/Cs_4_PbBr_6_ QDs with hexafluorobutyl methacrylate enabled the use of aqueous medium for the CO_2_R (Mu et al., 2019). Furthermore, to protect PQDs from contamination and to hinder their erosion by organic solvents, PQDs were encapsulated with metal oxides and MOFs while applying for CO_2_R (Zhang et al., 2016; Xu et al., 2018). The size optimization of PQDs is significant to accomplish the enhanced photocatalytic CO_2_R. The large size of PQDs decreases the surface area, while the smaller size leads to the aggregation, which will affect the optical absorption and charge carrier’s separation and transport properties. The CO_2_R performance of four different size (3.8, 6.1, 8.5, and 11.6 nm) CsPbBr_3_ PQDs in ethyl acetate/water medium under the solar illumination for 8 h concluded that PQDs with 8.5 nm size yielded more CH_4_, CO, and H_2_ products (Hou et al., 2017) (Figures 2A–F). The crystalline phase of PQDs influence the CO_2_R performance such as CsPbBr_3_ PQDs with the cubic phase are more active than the orthorhombic phase counterparts (Guo et al., 2019). The sluggish catalytic reaction dynamics of PQDs are dealt by employing a conducting material with high electron extraction efficiency (Xu et al., 2017; Pan et al., 2019).

### MXene Quantum Dots

MXenes, a set of 2D materials represented by a general formula of M_n+1_X_n_T_x_ (n = 1–4; X = C, N, and C/N; T_x_ = -O, -F, -OH, etc.), have exhibited a great potential in various applications owing to their exceptional electrical conductivity, metal-terminated surfaces, and hydrophilic characteristics (Lim et al., 2020; Tang et al., 2021). DFT calculations predicted that the chemisorption of CO_2_ is favorable compared to water on the MXene surface and higher electrical conductivity of MXene could cause the photocatalytic CO_2_R (Tahir et al., 2021). MXenes can be synthesized by the selective chemical etching of “A” layers from their sandwich-like parent MAX phase precursors, consisting of a stacked MXene nanosheets separated by the layers of A group elements. Recently, it has been demonstrated that appropriate experimental conditions could fragment the 2D MXenes into tiny pieces (≤10 nm), known as MXene QDs (MQDs). MQDs inherit all characteristics of their 2D counterparts and exhibit additional unique properties emanating from their high surface area and quantum size effects. MQDs absorb light in the range of UV to NIR and capable of effectively transforming the absorbed light energy into other forms, including chemical energy. Furthermore, the smaller size and hydrophilic/reactive surface functional groups permit easy grafting on other semiconductor nanostructures to make heterostructures. Recently, a facile incorporation of Ti_3_C_2_ MQDs onto Cu_2_O nanowires (NWs)/Cu mesh (Ti_3_C_2_ MQDs/Cu_2_O/Cu heterostructure) through a self-assembly approach was demonstrated to improve the CO_2_R (Zeng et al., 2019) (Figures 2G–K). The grafting of MQDs has improved the stability Cu_2_O NWs and led to significant enhancement in CO_2_R performance by improving light absorption and inhibiting the charge recombination. Furthermore, the CH_3_OH yield obtained with the Ti_3_C_2_ MQDs/Cu_2_O NWs/Cu photocatalyst is 8.25 and 2.15 times higher than Cu_2_O NWs/Cu and Ti_3_C_2_ sheets/Cu_2_O NWs/Cu photocatalysts, respectively. As the Fermi level (E_F_) of Ti_3_C_2_ MQDs is less negative than the CB of Cu_2_O, photogenerated charge carriers migrate from Cu_2_O to Ti_3_C_2_ MQDs and accumulate. The E_F_ of MQDs is sufficiently negative to perform the reduction of CO_2_ to CH_3_OH, with accumulated electrons accelerating the CO_2_R. On the other hand, the E_F_ of MXene nanosheets is positive, which is not suitable for accelerating the CO_2_R.

## Conclusions and Perspectives

Photochemical CO_2_R is one of the efficient methods for the conversion of solar to fuel without releasing any toxic wastes into the environment. An ideal photocatalyst should have the qualities like a high surface area, more active sites, long-term stability, low cost, and easy to produce in industrial scale to commercialize. Several kinds of QDs such as MOQDs, TMCQDs, CQDs, PQDs, and MQDs have been studied so far for the photocatalytic CO_2_R. Overall, the research on QDs for CO_2_R is still in its infancy, and following aspects need to be addressed to reach further growth for the ease in applicability. The size control of most QDs involves complicated synthesis procedures. Developing a simple, cost-effective, size-controlled, and highly efficient synthesis approaches will lead to wide utilization of QDs for CO_2_R. Most of the TMCQDs and PQDs for CO_2_R are based on Cd- and Pb-containing compositions, respectively, which are not ideal in the perspective of safety and eco-friendliness. Hence, more research is needed for improving the stability and CO_2_R efficiency of Cd- and Pb-free QDs (such as InP, ZnSe, and ZnS) (Wang et al., 2019). The poor oxidation stability of the TMCQDs, PQDs, and MQDs in the presence of water and light are the major challenging aspects to be addressed immediately. The QD-based hybrids are mostly achieved by simple blending of QDs with other semiconductors, which does not generate a strong chemical interaction at the interfaces for efficient charge transfer process. Hence, *in situ* growth methodologies and/or external functionalization with different functional groups/molecules should be adopted to fully exploit the advantages of QDs. The full spectrum of solar light utilization by QDs for CO_2_R is not yet accomplished. More innovative technologies like making QDs comprising upconversion material as core should be investigated. At present, formic acid and CO are the main products of CO_2_R via two electron reduction. Hence, CO_2_R *via* four-, six-, and eight-electron reduction needs significant attention. Especially, methane production by eight-electron reduction can make a vital change in the application of QDs for CO_2_R. The H_2_ production via proton reduction that reduces the CO_2_R efficiency is another critical concern for the QD-based systems. Although the efficient properties of CQDs depends on the size, shape, surface defects, and heteroatom doping concentrations, a well-established method(s) for precise tuning is needed. Furthermore, the functional groups of CQDs are known to be reduced with the prolonged exposure of light during the photocatalytic reactions, which may influence their CO_2_R activity. Hence, improving the stability is a bottleneck concern in CQD-based CO_2_R research. The advancement in QDs stability under light, highly interactive interface with other materials, morphological control, and quick adoptability to the reaction environment will make them as futuristic materials for not only in CO_2_R but also in other interdisciplinary fields.
